# Symmetry-broken square silicon patches for ultra-narrowband light absorption

**DOI:** 10.1038/s41598-019-54003-6

**Published:** 2019-11-25

**Authors:** Xin Yin, Tian Sang, Honglong Qi, Guoqing Li, Xun Wang, Jicheng Wang, Yueke Wang

**Affiliations:** 1 Department of Photoelectric Information Science and Engineering, School of Science, Jiangnan University, Wuxi, 214122 China; 2 Jiangsu Provincial Research Center of Light Industrial Optoelectronic Engineering and Technology, Jiangnan University, Wuxi, 214122 China

**Keywords:** Metamaterials, Optical sensors, Sub-wavelength optics

## Abstract

The effect of ultra-narrowband light absorption enhancement is presented by using metamaterials with symmetry-broken square silicon patches (SSPs). The symmetry of the SSP can be broken by introducing a narrow slit deviating from its center. By breaking the symmetry of the SSPs, slit resonance mode with standing wave patterns can be excited, and the locations of the absorption peaks can be well estimated by using the Fabry-Pérot (F-P) cavity model. Although there is no excitation of surface plasmon resonance, ultra-narrowband light absorption can be achieved by minimizing the reflectance through perfect impedance matching and simultaneously eliminating the transmittance by the metallic substrate. Good ultra-narrowband absorption features can be maintained as the parameters of the buffer layer and the SSPs are altered. When this type of symmetry-broken SSPs-based metamaterial is used in refractive-index sensors, it shows excellent sensing properties due to its stable ultra-narrowband absorption enhancement.

## Introduction

Light absorption enhancement are drawing significant interest owing to its importance in both science and practical applications. In many applications especially in the fields such as thermal radiation tailoring^1^, sensitive detection^2,3^, and sensing^4,5^, narrowband light absorption enhancement is particularly desirable. In the past decade, hybrid metal/dielectric systems such as the metal-dielectric-metal (MIM) sandwich structures have been intensively investigated due to the nearly-perfect absorption or perfect absorption performances^6–18^. These MIM structures usually consist of periodically arranged metal patches and a metallic substrate separated by a dielectric layer, the electric resonance can be generated by the metal patches and the magnetic resonance can be produced by the anti-parallel currents in the top and bottom metal layers^6^. By using different shapes of metal patches such as stripe^7,8^, rectangular (square)^9–11^, cross^12,13^, circle^14–16^, and ring^17,18^, incident light can be absorbed via the resonant cavity modes which are resulted from the coupled surface plasmon resonance in the upper and lower metal-dielectric interface. However, the absorption bandwidths of the MIM structures are typically above tens of nanometers, and it is difficult to further reduce their bandwidths due to the inherent Ohmic losses in both the top and bottom metal layers.

Recently, new strategies based on symmetry breaking have been developed and shown great promise for reducing the bandwidth of the resonator system^19,20^. In this approach, the resonator system is generally designed to support the bright and dark resonances. By introducing symmetry breaking in the structural shape, a weak coupling between the two resonances with the features of Fano resonances can be excited, which leads to extended resonance lifetimes and spectrally narrow lineshapes^21–23^. In addition, symmetry breaking of the resonator system with symmetrical structural shape can also be realized at off-normal incidence^24,25^. The introducing of off-normal incidence will break the symmetry of the incident light, which permits the coupling between the incident plane wave and modes that are inaccessible at normal incidence due to a mismatch in their symmetries, thus narrow resonance peaks can be achieved due to the excitation of symmetry-protected modes^26–29^. To date, the strategies of symmetry-breaking have attracted increasing interests due to their diverse optical functions for various applications, such as transmission filters^27–31^, high quality resonance^32–35^, plasmon-induced transparency^36–39^, directional couplers^40^, second-order nonlinear effects^41,42^, chiral metamaterials^43,44^, and plasmon mode splitting^45,46^. However, there are only few researches on narrowband light absorption enhancement by using the symmetry-broken metamaterials.

In the present paper, metamaterial with symmetry-broken square silicon patches (SSPs) is proposed to achieve ultra-narrowband light absorption enhancement. The high-index SSPs adhere to a metallic substrate separated by a low-index buffer layer, and the symmetry of the SSPs can be broken by introducing a narrow slit which is deviated from the center of the SSP. It is shown that the slit resonance with highly confined electric-field can be excited by the symmetry breaking of the SSPs, and the locations of the absorption peaks can be well estimated by using the Fabry-Pérot (F-P) cavity model. Although there is no excitation of surface plasmon resonance, by using the symmetry breaking of the SSPs, ultra-narrowband light absorption with the bandwidth of 0.2 nm can be obtained by minimizing the reflectance through perfect impedance matching and simultaneously eliminating the transmittance by the metallic substrate. Good ultra-narrowband absorption features can be maintained as the parameters of the buffer layer and the SSPs are altered. Moreover, this type of ultra-narrowband absorber shows excellent refractive-index sensing performances, and sensitivity as high as 405 nm/RIU with the figure of merit (FOM) of 2025 and FOM^*^ = 93580 can be obtained in the near-infrared wavelength region.

## Design Principles

**Figure 1:**
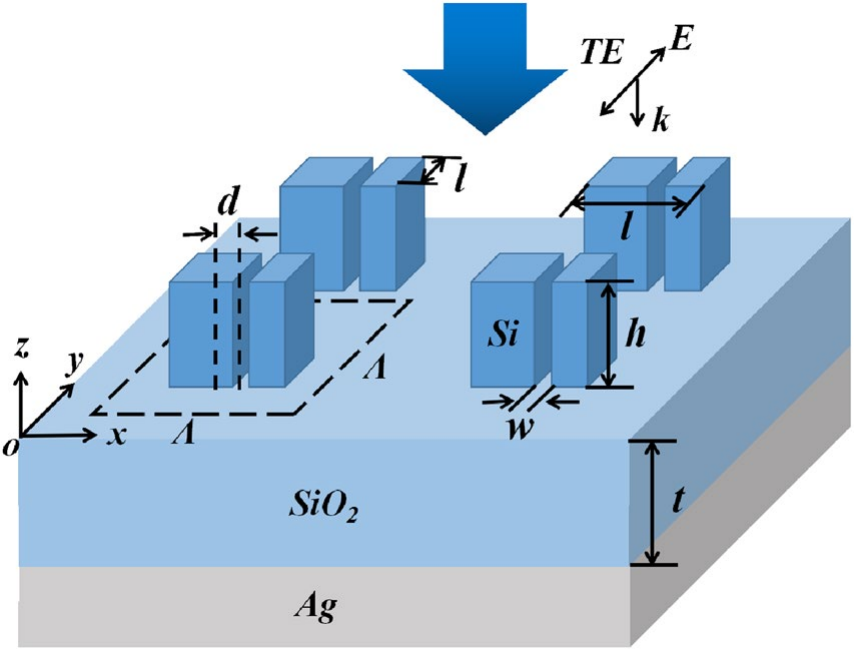
Schematic diagram of the SSPs-based metamaterial with narrow slits illuminated by normal incident TE wave.

Figure 1 shows schematic diagram of the SSPs-based metamaterial with narrow slits under TE wave illumination (electric-field vector lies along the *y*-axis). From the top to the bottom are periodic high-index SSPs with narrow slits, a low-index SiO_2_ buffer layer with the thickness of *t*, and a semi-infinite Ag substrate, respectively. The background is air with refractive-index equal to 1. In the near-infrared wavelength region, the refractive-index of silicon is 3.48, the refractive-index of SiO_2_ is 1.47, and the frequency-dependent refractive-indexes of the Ag film are taken from Palik^47^. The period of the unit cell is *Λ*, the length and height of the SSP are indicated as *l* and *h*, respectively. To evaluate the absorption performances of the SSPs-based metamaterial with symmetrical or asymmetrical profiles, a narrow slit with a width of *w* is introduced in the SSP, the distance between the center of the narrow slit and the center of the SSP is *d*. The structure of the SSPs is symmetric as *d* = 0, and the structural symmetry of the SSPs is broken as *d* ≠ 0. In simulation, three-dimensional finite-difference time-domain (FDTD) models of the commercial software FDTD Solutions are used to analyze the absorption properties of the SSPs-based metamaterial, and the absorption can be simplified as A = 1 − R, where R is reflection, which is because the transmission channel is blocked by the optically thick Ag film.

**Figure 2:**
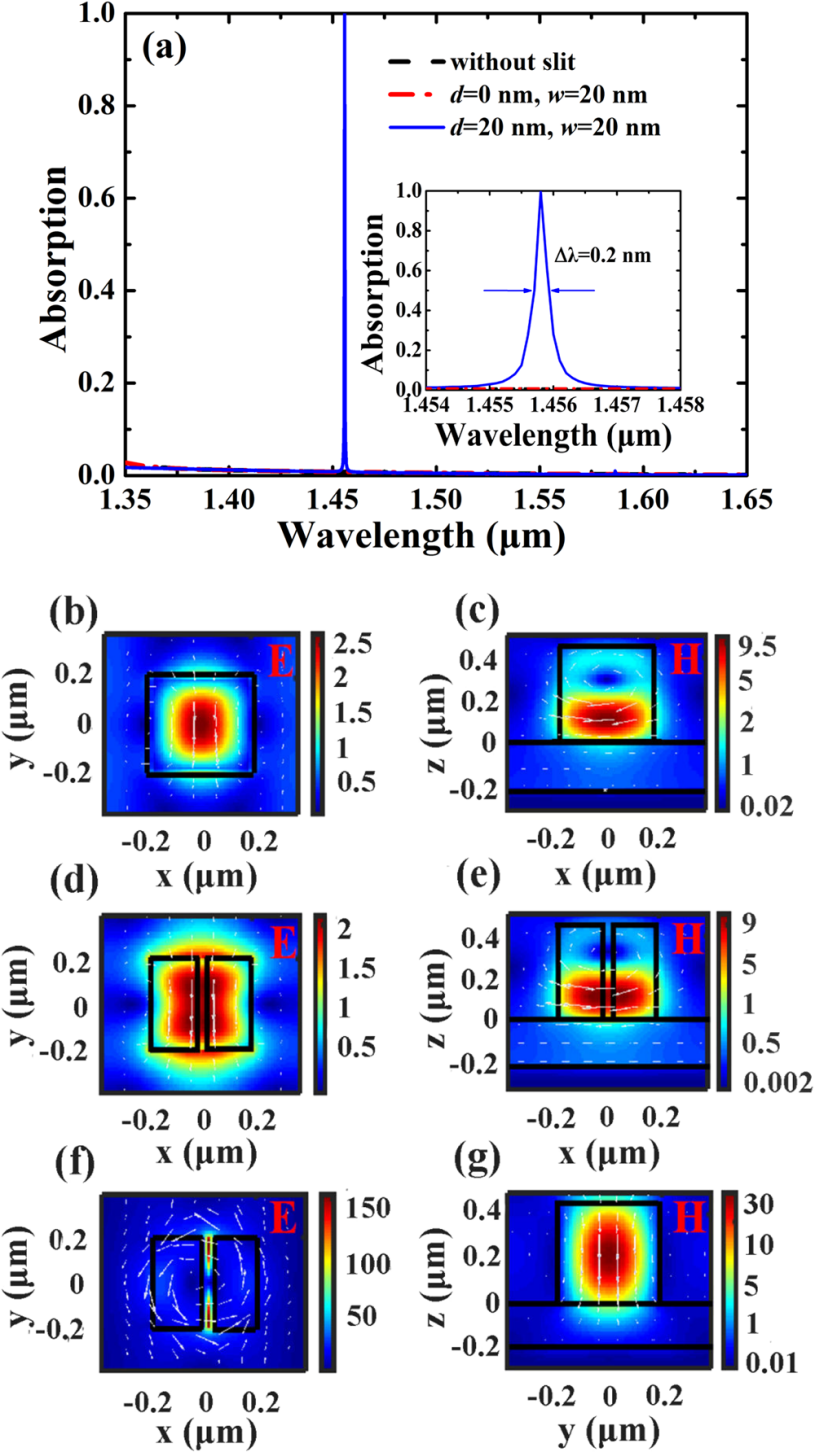
(**a**) Absorption response of the SSPs-based metamaterial for the symmetrical (without slit; or *d* = 0 nm, *w* = 20 nm) and symmetry-broken (*d* = 20 nm, *w* = 20 nm) structures. The inserted figure is the zoomed in view of the absorption response of the symmetry-broken structure. (**b**) and **(c)** are normalized electric-field and magnetic-field distributions of the SSPs without slit at the center of silicon patch at 1455.8 nm, respectively. (**d**) and **(e)** are normalized electric-field and magnetic-field distributions of the SSPs with *d* = 0 nm and *w* = 20 nm at the center of silicon patch at 1455.8 nm, respectively. **(f)** Normalized electric-field distributions of the symmetry-broken SSPs at the center of silicon patch at 1455.8 nm. **(g)** Normalized magnetic-field distributions of the symmetry-broken SSPs at the center of slit at 1455.8 nm. White dashed lines and arrows indicate field direction.

Figure 2 shows absorption performances and electric-field distributions of the SSPs-based metamaterial for the symmetrical and symmetry-broken structures. The structural parameters are: *Λ* = 780 nm, *l* = 400 nm, *h* = 480 nm, and *t* = 200 nm. As can be seen in Fig. 2a, there is no absorption enhancement for the SSPs-based metamaterial with symmetrical structures (without slit; or *d* = 0 nm, *w* = 20 nm). This is because the high-index silicon patches with symmetrical profiles can be functioned as a broadband mirror with very high reflectivity (>99%) in the near-wavelength region^48,49^, and the incident light will be totally reflected into the background. However, for the symmetry-broken SSPs, an extraordinarily resonant peak with near-perfect absorption (A = 99.9%) is occurred at 1455.8 nm with the bandwidth of Δλ = 0.2 nm, and there is no light absorption enhancement as wavelength is deviated from the resonance wavelength.

To provide a physical understanding of the phenomenon of anomalous resonance absorption in Fig. 2a, the electric and magnetic field patterns of the symmetrical and symmetry-broken SSPs at resonant peak are plotted in Fig. 2b–g. As can be seen in Fig. 2b, the electric-field distribution at 1455.8 nm is highly confined by the silicon patch for the symmetrical SSPs without a slit, and its near fields are similar to those of an electric dipole oriented along the *y* direction, indicating the properties of the electric resonant mode^50–52^. This is not surprising since there is a magnetic loop associated with the electric dipole in the *x*-*z* plane seen in Fig. 2c. In Fig. 2d,e, it can be seen that the electric resonant mode is also excited for the symmetrical SSPs with a narrow slit. However, as shown in Fig. 2f,g, for the symmetry-broken SSPs with a narrow slit, one can observe that the near-field patterns of the SSPs are very similar to those of a magnetic dipole oriented along the *z* direction, and a slit resonance mode with standing wave patterns along the *y* direction is excited. In general, both the electric and magnetic dipole resonances can be excited as light hits a high-index dielectric particle, making the particle behave like a magnetic dipole (first Mie resonance) and an electric dipole (second Mie resonance)^53–55^. For the structure of the symmetry-broken SSPs, the electric-field energy is transferred from the silicon patch to the narrow slit due to the magnetic dipole resonance, and electric hotspots are occurred at both ends of the slit along the *y* direction. The maximum of the normalized electric-field amplitude of the resonant peak is 150, which significantly enhance the light intensity to 22500 times. The greatly enhanced electric-field can effectively alter the near-field distributions of the surface of the SSPs-based metamaterial, which is directly related to the asymmetry-driven absorption enhancement.

**Figure 3:**
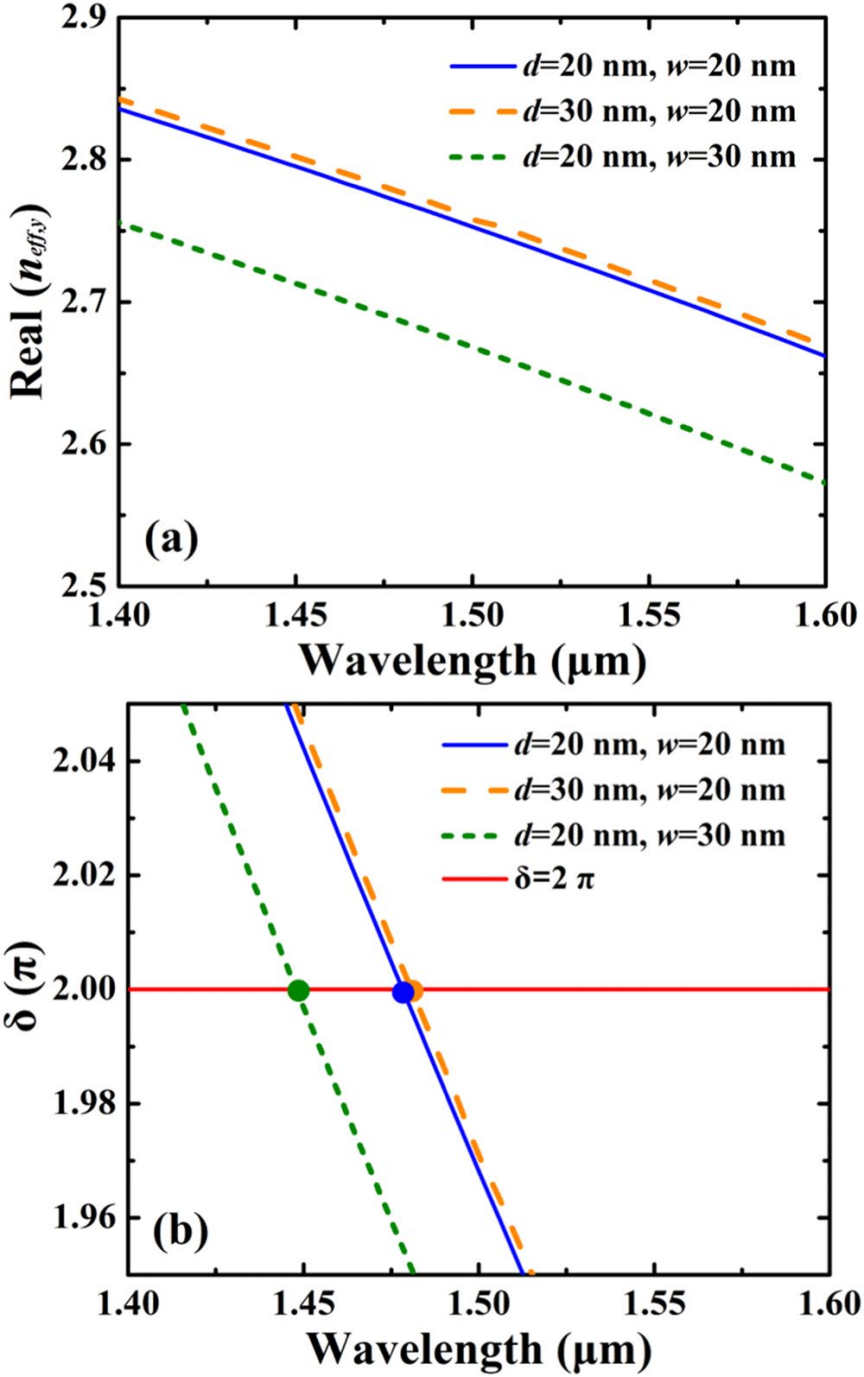
(**a**) Real part of the effective refractive-index Re(*n*_*eff,y*_) of the slit resonance mode of the SSPs vs. wavelength under different location *d* and width *w* of the slit. (**b**) Resonance wavelengths estimated by using the F-P cavity model for different location *d* and width *w* of the slit.

For the symmetry-broken SSPs, since the light field energy of the slit resonance mode exhibits the standing wave resonance characteristics in the *y* direction, we initially conducted the mode analysis of the slit resonance under different locations and widths of the slit. In calculation, the structural parameters are: *Λ* = 780 nm, *l* = 400 nm, *h* = 480 nm, and *t* = 200 nm. The effective refractive-indices of the slit resonance mode in the *y* direction *n*_*eff,y*_ ≡ *k*_*y*_/*k*_0_ are obtained by using finite-element method (FEM) with the software COMSOL (where *k*_0_ is the wave vector in free space and *k*_*y*_ is the wave vector along the *y* axis)^56^. Figure 3a shows Re(*n*_*eff,y*_) as a function of wavelength for different location *d* and width *w* of the slit. As can be seen in Fig. 3a, Re(*n*_*eff,y*_) is decreased with the increase of wavelength for different *d* and *w*. In addition, Re(*n*_*eff,y*_) can be significantly affected by altering *d* and *w*, Re(*n*_*eff,y*_) is reduced as *w* is increased or the symmetry of the structure is increased (i.e. *d* is decreased). For a fixed wavelength, Re(*n*_*eff,y*_) will be the maximum for the smallest *w* and the largest *d*. Here, the slit resonance can be equivalent to a localized F-P cavity resonance due to the standing wave patterns along the *y* direction, and the F-P cavity resonance condition can be described as^57,58^:

1 $$\delta =\frac{2\pi l{\rm{Re}}({n}_{eff,y})}{\lambda }+\phi =m\pi $$

where *δ* is the phase shift, *λ* is the resonance wavelength, *l* = 400 nm is the F-P cavity length, *ϕ* = *π/2* is the phase shift, *m* is an integer which represents the resonant order, and *m* = 2 in calculation.

Figure 3b shows the resonance wavelengths of the symmetry-broken SSPs estimated by using the F-P cavity model for different locations and widths of the slit. The structural parameters are the same as in Fig. 3a. As shown in Fig. 3b, the resonance wavelengths calculated by using the F-P cavity model for (*d* = 20 nm, *w* = 30 nm), (*d* = 20 nm, *w* = 20 nm) and (*d* = 30 nm, *w* = 20 nm) are 1447.5 nm, 1476.4 nm and 1481.2 nm, respectively. The wavelengths of resonant absorption calculated by using the FDTD method for (*d* = 20 nm, *w* = 30 nm), (*d* = 20 nm, *w* = 20 nm) and (*d* = 30 nm, *w* = 20 nm) are 1408.3 nm, 1455.8 nm and 1464.3 nm, respectively. The results of the F-P cavity model are in good agreement with those of the FDTD, and the maximum of the relative errors of the F-P cavity model is less than 2.8%. Thus the simplified F-P model can provide reliable estimation of the locations of absorption peaks of the SSPs-based metamaterial.

To better understand the physical basis of the ultra-narrowband light absorption enhancement of the SSPs-based metamaterial, the input impedances of the structure are studied by using the impedance theory. According to the impedance theory^59,60^, the relation between the scattering parameters and impedance *Z* can be expressed as:

2 $${S}_{21}={S}_{12}=\frac{1}{\cos ({\rm{N}}k{\rm{H}})-\frac{i}{2}(Z+\frac{1}{2})\sin ({\rm{N}}k{\rm{H}})}$$

3 $${S}_{11}={S}_{22}=\frac{i}{2}(\frac{1}{Z}-Z)\sin ({\rm{N}}k{\rm{H}})$$

where *S*_11_, *S*_21_, *S*_12_, *S*_22_ are scattering parameters, *N* is the effective refractive-index of the structure, *H* = *h* + *t*, and *k* the wave vector. The input impedance *Z* of the structure can be written as $Z=\pm \sqrt{[{(1+{S}_{11})}^{2}-{S}_{21}^{2}]/[{(1-{S}_{11})}^{2}-{S}_{21}^{2}]}$, and the reflection of the SSPs-based metamaterial can be calculated as $R={[(Z-{Z}_{0})/(Z+{Z}_{0})]}^{2}$, where ${Z}_{0}=\sqrt{\mu (\omega )/\varepsilon (\omega )}=1$ is the impedance of the free space. Obviously, the impedance of the structure should be well matched with that of the free space so as to reduce reflection.

**Figure 4:**
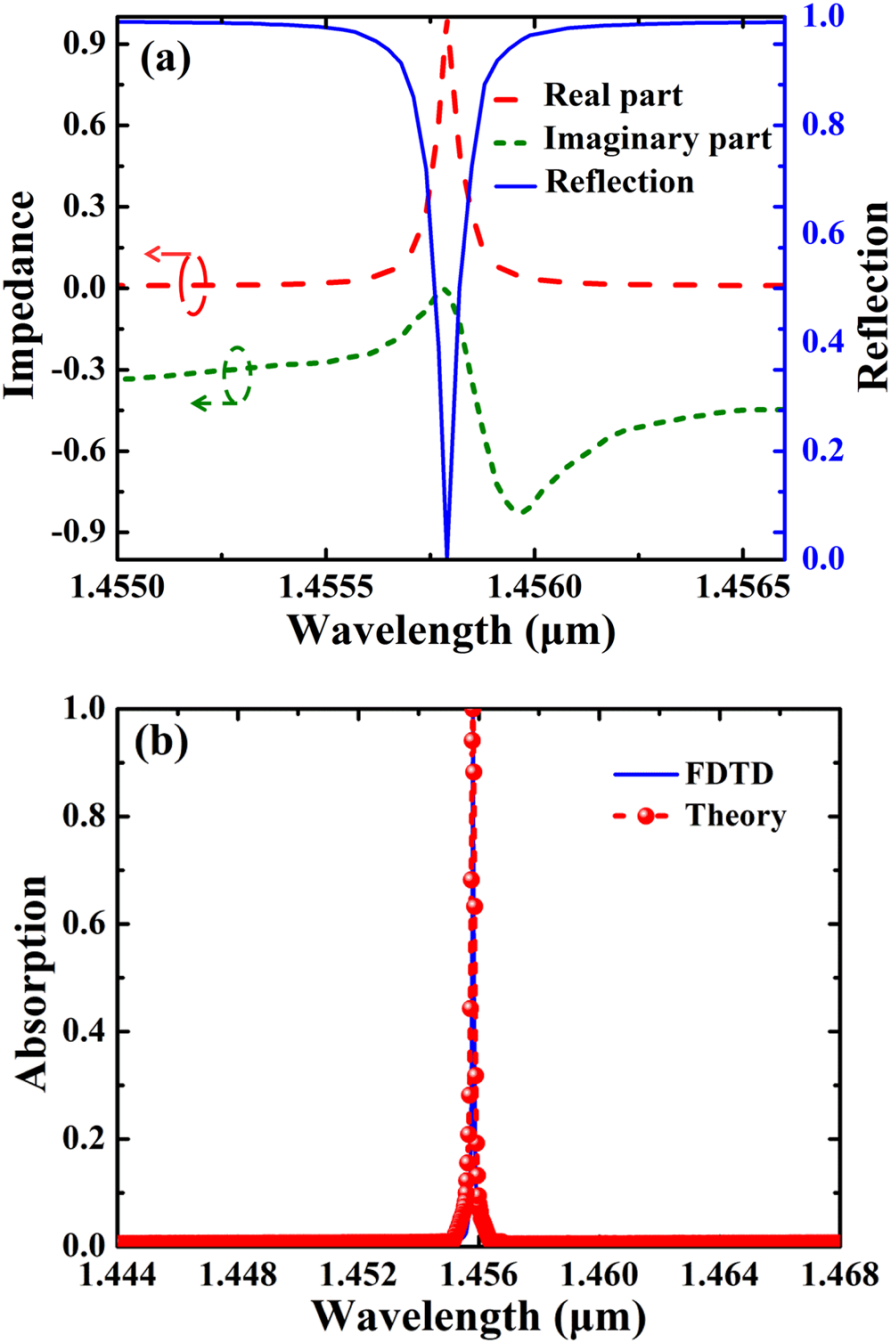
(**a**) Input impedance and reflection response of the SSPs-based metamaterial. (**b**) FDTD result and theoretical result of absorption spectrum of the SSPs-based metamaterial.

Figure 4a shows the input impedance and reflection response of the SSPs-based metamaterial, the parameters are the same as those in Fig. 2a with *d* = 20 nm and *w* = 20 nm. As can be seen in Fig. 4a, both the real and imaginary parts of *Z* are varied abruptly around the absorption peak so as to meet the impedance matching condition. In particular, the real part of *Z* approaches 1, and the imaginary part of *Z* tends to 0, and perfect impedance matching condition can be achieved at the resonance wavelength, which confirms the preceding theoretical analysis on perfect absorption enhancement of the SSPs-based metamaterial. Note the absorption mechanism of the SSPs-based metamaterial is complete different from that of the conventional MIM-based absorbers^6–18^ because there is no excitation of surface plasmon resonance under the TE wave illumination; however, perfect absorption enhancement can be realized by minimizing the reflectance through perfect impedance matching and simultaneously eliminating the transmittance by the Ag substrate. Figure 4b shows the theoretical and FDTD results of the absorption response of the SSPs-based metamaterial. The theoretical results of the absorption response are calculated by using the retrieved input impedances, which show good agreement with those of the FDTD.

## Results and Discussion

**Figure 5:**
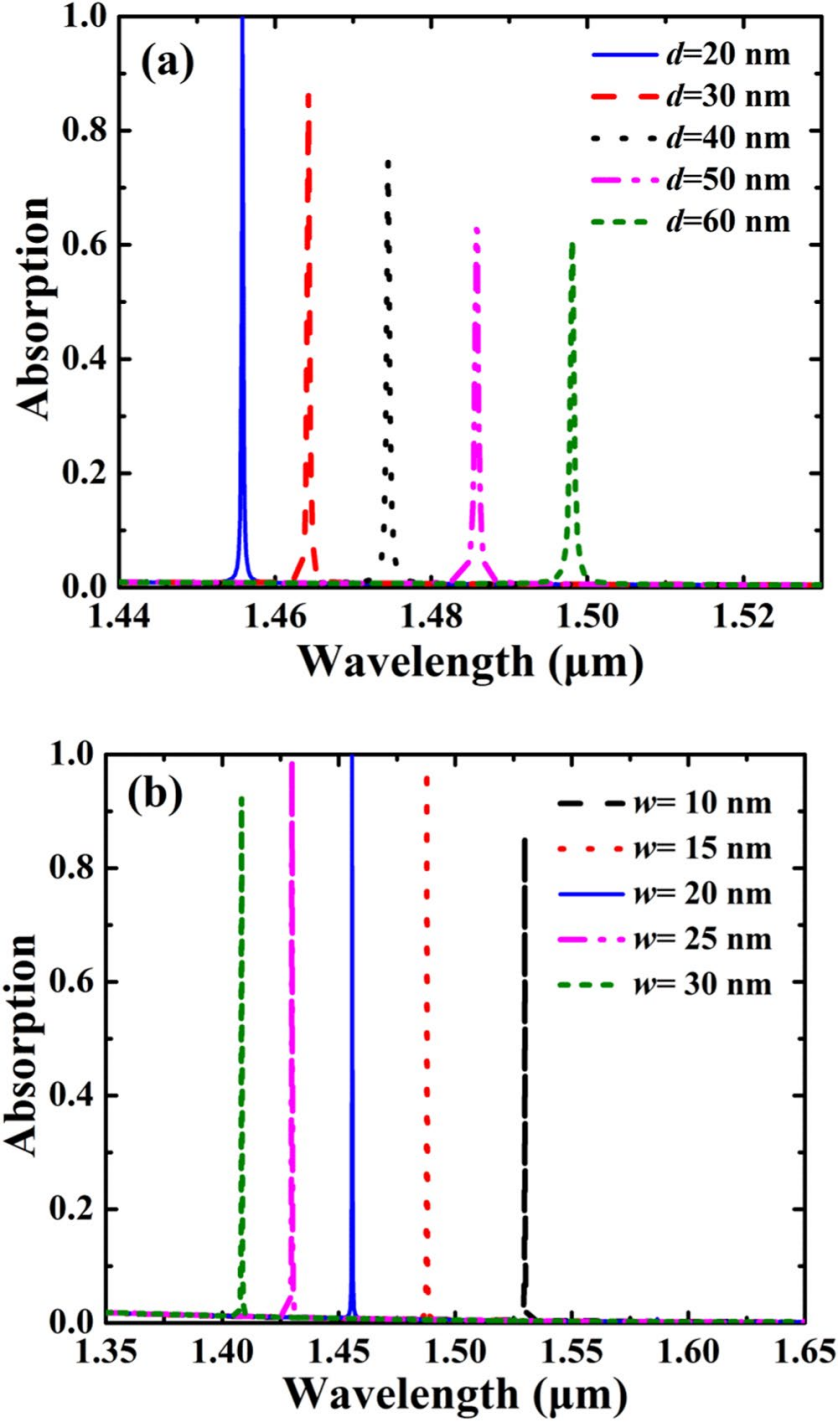
Influence of the structural parameters of the slit on absorption spectra of the SSPs-based metamaterial. The parameters are: *Λ* = 780 nm, *l* = 400 nm, *h* = 480 nm, and *t* = 200 nm. (**a**) Absorption spectra as a function of the location of the slit with *w* = 20 nm. (**b**) Absorption spectra as a function of the width of the slit with *d* = 20 nm.

To further evaluate the selective absorption performances of the proposed structure, we first investigated the influence of the structural parameters of the slit on absorption spectra of the SSPs-based metamaterial. Figure 5 shows influence of the location and the width of the slit on the absorption spectra of the symmetry-broken SSPs-based metamaterial. As can be seen in Fig. 5a, ultra-narrowband absorption enhancement can be maintained as the location of the slit is significantly altered, and the absorption peak is red-shifted from 1455.8 nm to 1498.1 nm as *d* is increased from 20 nm to 60 nm. The red-shift of the absorption peak can be explained by the F-P cavity theory. As shown in Fig. 3a, the real part of effective refractive-index Re(*n*_*eff,y*_) of the slit is increased as *d* is increased, thus the absorption peak is red-shifted with the increase of *d* according to Eq. (1). Note the intensity of the absorption peak is slightly reduced with the increase of *d*, which is because that the normalized electric-field amplitude of the slit is reduced as *d* is increased. Figure 5b shows absorption spectra as a function of the width of the slit with *d* = 20 nm. As can be seen in Fig. 5b, ultra-narrowband absorption enhancement can also be maintained as *w* is varied. The absorption peak is blue-shifted from 1529.9 nm to 1408.3 nm as w is increased from 10 nm to 30 nm, and the shift of the absorption peak is more obvious comparing with the variation of *d*. As shown in Fig. 3a, because Re(*n*_*eff,y*_) of the slit is more sensitive to the variation of *w* comparing with *d*, the variation of *w* will result in larger shift of the absorption peak.

**Figure 6:**
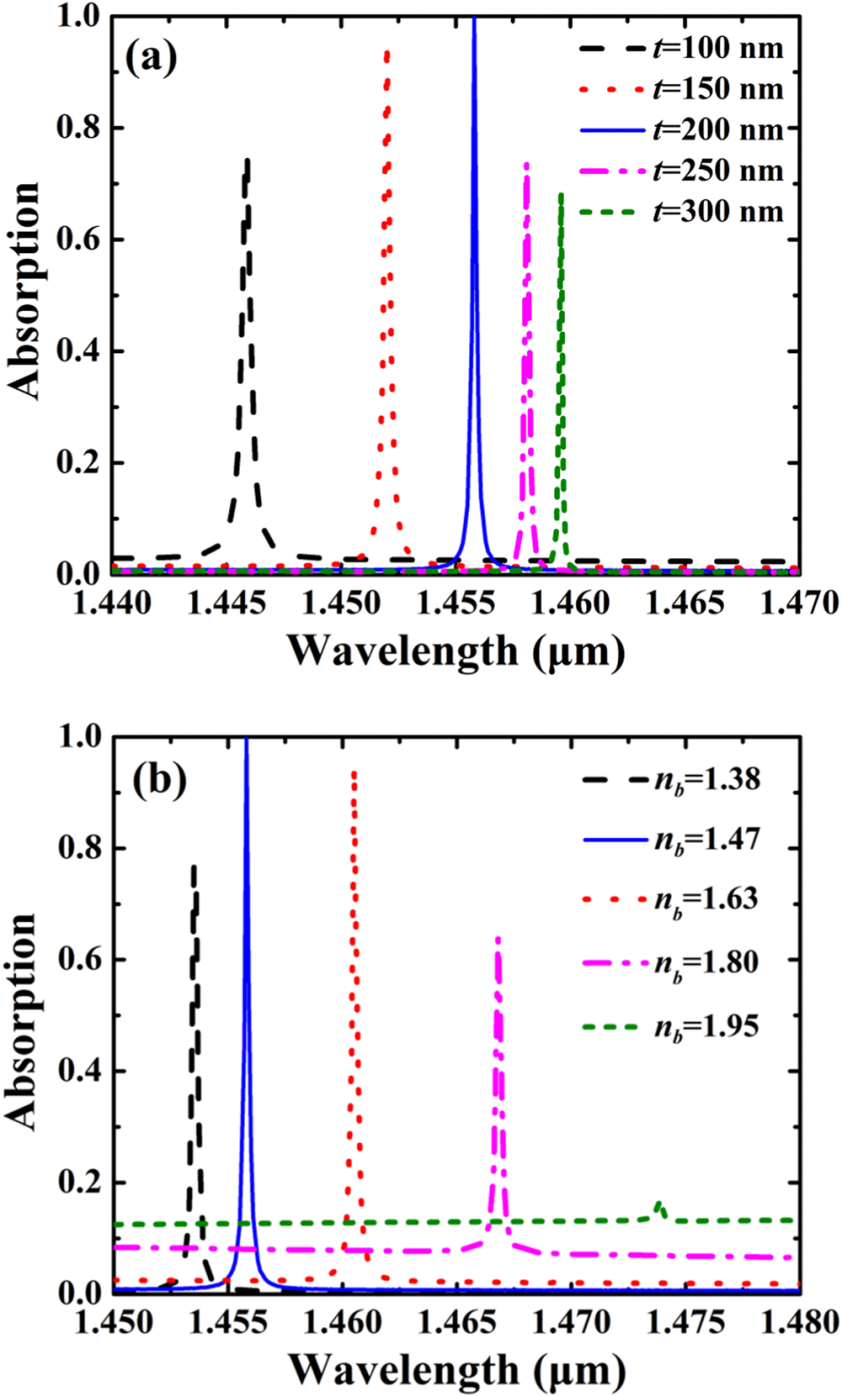
Influence of the structural parameters of the buffer layer on absorption spectra of the SSPs-based metamaterial. The parameters are: *h* = 480 nm, *d* = 20 nm, *w* = 20 nm, *l* = 400 nm, and *Λ* = 780 nm. (**a**) Absorption spectra as a function of *t* with *n*_*b*_ = 1.47. (**b**) Absorption spectra of as a function of refractive index *n*_*b*_ with *t* = 200 nm.

Figure 6 shows influence of the structural parameters of the buffer layer on absorption spectra of the SSPs-based metamaterial. According to Fig. 6, perfect absorption can be achieved at the specific parameters (*t* = 200 nm, *n*_*b*_ = 1.47) due to perfect impedance matching, and deviation from these parameters will result in the decrease of the peak absorption. As can be seen in Fig. 6a, the increase of the thickness of buffer layer will redshift the absorption peak. The increase of the thickness of the buffer layer will increase the equivalent optical thickness of the structure, thus the absorption peak is shifted to the longer wavelength as *t* is increased. As show in Fig. 6b, the absorption peak is redshifted with the increase of refractive index of the buffer layer, in particular, the absorption efficiency tends to vanish as the refractive index of the buffer layer is increased.

According to the effective-medium theory^61^, the equivalent refractive index of SSPs can be approximately estimated as:

4 $${n}_{equ}=\sqrt{\frac{[(1-F){n}^{2}+F{n}_{{\rm{Si}}}^{2}][F{n}^{2}+(1-F){n}_{{\rm{Si}}}^{2}]+{n}_{{\rm{Si}}}^{2}}{2[F{n}^{2}+(1-F){n}_{{\rm{Si}}}^{2}]}}$$

where *F* = *l*/*Λ* is the fill factor of the SSPs. According to Eq. (4), the equivalent refractive index of SSPs can be calculated as 2.07 for *l* = 400 nm and *Λ* = 780 nm. In Fig. 6b, as the refractive index of the buffer layer is increased to 1.95, the refractive index of the buffer layer approached the equivalent refractive index of SSPs, and the role of high-contrast SSP for supporting the highly-confined resonant mode is cancelled, resulting in the vanishment of the absorption peak.

Finally, we showed that the symmetry-broken SSPs-based metamaterial can be functioned as a refractive-index sensor due to its robust ultra-narrowband absorption performances. In a sensing application, the sensing capability is usually defined as follows^62–64^:

5 $$S=\frac{\Delta \lambda }{\Delta {\rm{n}}},\,{\rm{FoM}}=\frac{S}{FWHM},\,{{\rm{FoM}}}^{\ast }={(\frac{\Delta I/\Delta n}{I})}_{{\rm{\max }}}$$

where Δ*λ* is the peak wavelength change with the refractive-index change Δ*n*, and *FWHM* is the half-width of the absorption band. The ΔI/I is the relative absorption intensity change at the fixed wavelength which is chosen for a maximum FOM^*^. FoM^*^ shows the whole sensitivity capability when measuring the reflected light intensity for one particular wavelength.

**Figure 7:**
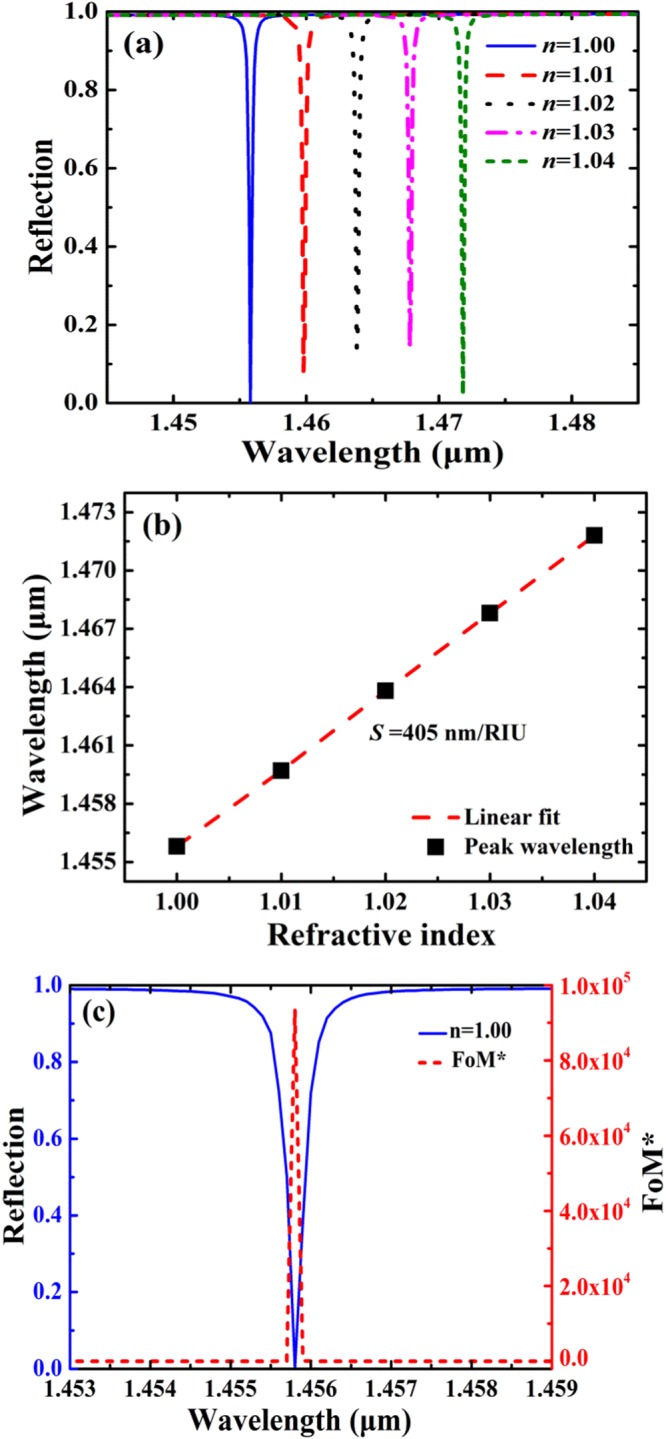
Sensing properties of the SSPs-based metamaterial. The parameters are: *Λ* = 780 nm, *l* = 400 nm, *d* = 20 nm, *w* = 20 nm, *h* = 480 nm, and *t* = 200 nm. (**a**) Reflection spectra as a function of refractive-index of the background. (**b**) Spectral position of the absorption peak as a function of refractive-index of the background. (**c**) Reflective spectrum (*n* = 1.00) and FoM^*^ curve.

Figure 7a shows reflection spectra as a function of refractive-index of the background. As can be seen in Fig. 7a, the absorption peak is red-shifted with the increase of the refractive-index of the background. The absorption peak is shifted from 1455.8 nm to 1471.8 nm as the refractive-index of the background is increased from 1.00 to 1.04. In Fig. 7b, by using a linear fit, the simulation has a predicted sensitivity of 405 nm/RIU, where RIU is per refractive-index unit, and a high FoM of 2025 can be achieved. By using the reflection data as inputs, FoM^*^ can be calculated by using Eq. (5) and the calculated results are shown in Fig. 7c. It is shown that FOM^*^ reaches a high value of 93580 at the resonant wavelength of 1455.8 nm. The sensitivity of 405 nm/RIU of the SSPs-based metamaterial is comparable with many nanostructured optical sensors, such as plasmonic absorbers^4,56,62–64^ and guided-mode resonance gratings^65–67^. In addition, the FoM and FoM^*^ the SSPs-based metamaterial can be significantly higher than these nanostructured sensors^4,14,56,62–67^ due to its ultra-narrowband absorption performances. Therefore, ultra-narrowband absorption enhancement of the SSPs-based metamaterial may be suitable for high-performance sensor applications.

## Conclusion

In conclusion, ultra-narrowband light absorption enhancement can be realized with the absent of surface plasmon resonance by breaking the symmetry of the SSPs. The symmetry of the SSPs can be broken as the location of the narrow slit is deviated from the center of the SSP. Slit resonance mode can be excited by the symmetry-broken SSPs, and the locations of the absorption peaks can be well estimated by using the simplified F-P model. By breaking the symmetry of the SSPs, ultra-narrowband light absorption with the bandwidth of 0.2 nm can be achieved by minimizing the reflectance through perfect impedance matching and simultaneously eliminating the transmittance by the metallic substrate. Good ultra-narrowband absorption features can be maintained as the parameters of the buffer layer and the SSPs are altered. In addition, the symmetry-broken SSPs-based metamaterial shows excellent sensing properties due to its stable ultra-narrowband absorption performances. S, FoM, and FoM^*^ are 405 nm/RIU, 2025, and 93580, respectively. The strategy of breaking the symmetry to enhance light absorption can be generalized for designing more sophisticated nanostructures, which may be found applications in the ultrasensitive sensing and imaging systems in the near-infrared region.

## Methods

### Simulation of absorption response

In simulation, three-dimensional finite-difference time-domain (FDTD) approach is adopted to calculate the absorption performance of the SSP using a commercially available software package (Lumerical FDTD Solutions Inc.v8.19.1584). The reflection of the SSP is defined by the ratio of the reflected power to the launched power. Periodic boundary conditions are used in the *x* and *y* direction, and perfectly matched layer boundary conditions are used in the *z* direction. The grid size is chosen as 1 nm in all directions. The *x*, *y* and *z* directions and wave polarization are defined in Fig. 1.

### Calculation of effective refractive-indices

The effective refractive-indices of the slit resonance mode of the symmetry-broken SSP is calculated by using finite-element method (FEM). The numerical calculations were carried out using two-dimensional module of wave optics of the commercial software COMSOL (COMSOL Multiphysics 5.4). The symmetry-broken SSP was sub-divided into very small but finite size meshes, and each mesh is governed by a set of characteristic equations which describe its physical properties and boundary conditions. Although there is no general closed form solution of the effective refractive-indices with variation in wavelength, the equations of the meshes can be solved as a set of simultaneous equations to compute the effective refractive-indices of the slit resonance mode supported by the symmetry-broken SSP. In simulation, the effective refractive-indices of the slit resonant mode was calculated with respect to wavelength with a step size of 5 nm. A fine mesh of triangular elements with a maximum size of 100 nm was used for entire calculations, and scattering boundary conditions were used to avoid field reflections at the computational boundaries.
